# Editorial: “Source-tracking,” molecular epidemiology and antigenic diversity of SARS-CoV-2 infections causing coronavirus disease 2019, COVID-19

**DOI:** 10.3389/fgene.2022.1031750

**Published:** 2022-10-25

**Authors:** Charles A. Narh, Cordelia Manickum, Bathri Vajravelu, Davide Zella, Lydia Mosi

**Affiliations:** ^1^ Department of Medicine, and Royal Melbourne Hospital, The University of Melbourne, Parkville, VIC, Australia; ^2^ Department of Infectious Diseases, Monash University, Melbourne, VIC, Australia; ^3^ School of medicine, Duke University, Durham, NC, United States; ^4^ Department of Physician Assistant Studies at MCPHS University, Boston, MA, United States; ^5^ School of medicine, University of Maryland, Baltimore, MD, United States; ^6^ Department of Biochemistry, Cell and Molecular Biology, University of Ghana, Legon, Ghana

**Keywords:** COVID-19, Epidemiology, SARS-CoV-2, Evolution, Phylogenetics, Serosurveillance, Diagnostics

## Introduction to the Research Topic

In December 2019, Chinese Health Authorities reported the first cases of COVID-19, caused by SARS-CoV-2, to the WHO Country Office in China (Zhu et al.), noting a spectrum of symptoms from mild to severe pneumonia. Following global spread of the virus, the World Health Organization (WHO), on 30 January 2020, declared COVID-19 a Public Health Emergency of International Concern (Jee, 2020). As of 22 August 2022, there have been more than 590 million cases and over 6.4 million deaths due to the SARS-CoV-2 pandemic, with several thousands of cases being confirmed daily across the WHO regions (WHO, 2022). This Research Topic sought to publish both research and review articles on the epidemiology, population genetics, antigenic diversity, transmission dynamics and evolution of SARS-CoV-2.

We, the editorial team, encouraged submissions from different disciplines of SARS-CoV-2 research, and recommended 36 articles for publication following at least two peer-reviews. We hope these articles, briefly summarised with their major findings, appeal to the wider readership of the scientific community, to inform further research, clinical practice and public health control of the COVID-19 pandemic.

## Thematic areas of the research topic

**FIGURE 1:**
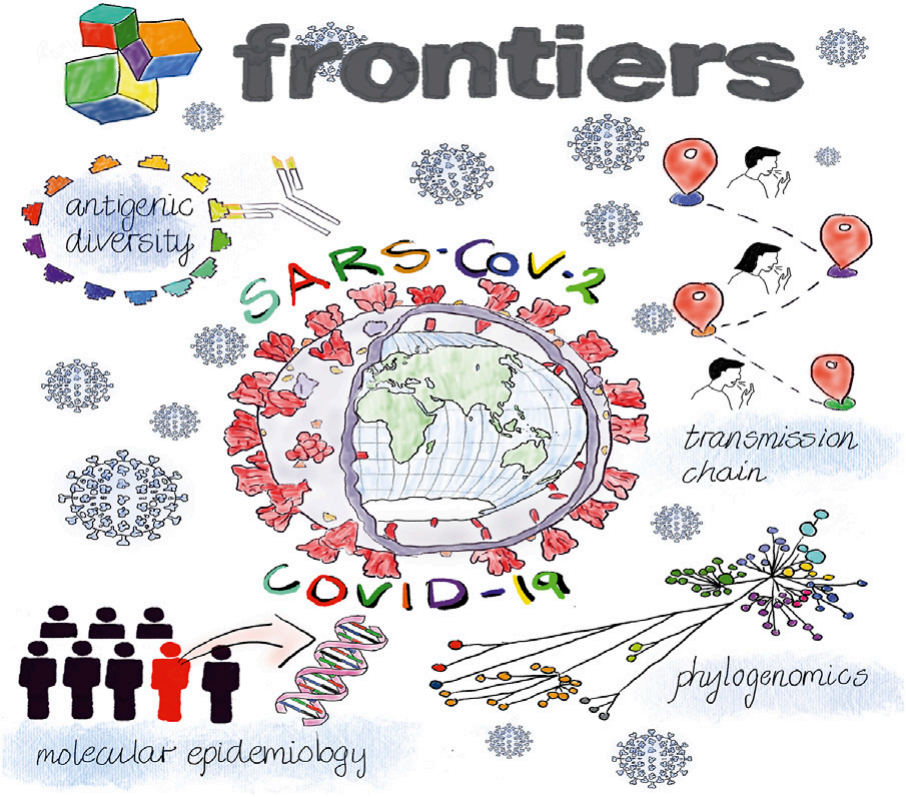
The infographic image for the research topic was created by Thorey Jonsdottir (Burnet Institute for Medical Research, Melbourne, Australia).

The articles published in this Research Topic were grouped into nine thematic areas—point-of-care diagnostics; clinical features and treatments; genomic surveillance and evolution, sero-surveillance and immunity; host-genetics; infection models; modelling COVID-19; Software programs for tracking cases; control and prevention. These themes fit within the remits of the research topic—“*Source-tracking, molecular epidemiology and antigenic diversity of SARS-CoV-2 infections causing COVID-19*” (Figure 1).

### Point-of-care diagnostic tools for COVID-19

Sakthivel et al. reviewed the literature and discussed molecular and serological assays that could be utilised for point-of-care (PoC) diagnosis of COVID-19. In particular, the authors proposed isothermal amplification techniques including loop mediated isothermal amplification (LAMP), which has comparable sensitivity and specificity as PCR, for the detection of SARS-CoV-2 infections in all respiratory sample types. Hofman et al. (2022) developed Ion Ampliseq, a next generation sequencing (NGS) technique, platform to genotype SARS-CoV-2 infections at the point-of-care, in an Academic Hospital Center in France (LPCE, Louis Pasteur Hospital, Nice, France). The authors further demonstrated the compatibility of this platform with nasopharyngeal and saliva samples containing low viral load.

### Clinical features and treatment of COVID-19

SARS-CoV-2 infections cause different clinical disease phenotypes ranging from asymptomatic to mild and severe/critical disease, with considerable fatality among vulnerable patients including the elderly and patients with underlying comorbidities. Zeng et al. compared four disease phenotypes—symptomatic cases (with or without pneumonia) and asymptomatic cases (with or without pneumonia). The authors noted that the group that was symptomatic with pneumonia consumed more medical resources than the other groups, cautioning for active monitoring in this group. The asymptomatic COVID-19 cases presented a similar viral load and viral shedding duration as the symptomatic COVID-19 cases.

Liu et al. also observed that COVID-19 patients with comorbidities were older, especially those with chronic obstructive pulmonary disease (COPD) and cardiovascular disease (CVD). Hypertension, diabetes mellitus, COPD, chronic kidney disease and CVD were mainly found in severe COVID-19 patients, indicating that age and the presence of comorbidities were risk factors for progression and poor prognosis in patients with COVID-19. To better understand how severe/critical disease was fatal in some patients, Chen et al. conducted a two-centre study, monitoring the clinical features among patients with severe disease. Using predictive models, the authors found lactate dehydrogenase, lymphocytes, procalcitonin, D-dimer, C-reactive protein, respiratory rate, and white-blood cells to be early predictors of mortality. Their findings indicated that clinical monitoring and interventions may be needed to lower the fatality among patients with severe disease.

The role of nutrition in gastrointestinal illness, in COVID-19, has been linked to gastrointestinal dysbiosis due to a loss of bacterial diversity, in particular of “beneficial” bacteria. Devaux et al. examined two major models: first, a virus directly causing damage locally (e.g., by inducing apoptosis of infected enterocytes); secondly, the indirect effect of the virus (e.g., by inducing changes in the composition of the gut microbiota followed by the induction of an inflammatory process). They suggested that both situations probably occur simultaneously in COVID-19 patients. Devaux et al. further suggested that maintaining a balanced immune response in COVID-19 was essential to improve patient outcome. To reduce the intestinal proinflammatory states in COVID-19 patients, they proposed the addition of nutritional supplements—butyrate (4 g sodium butyrate daily), L-tryptophan (4 mg/kg of body weight daily) and Vitamin D3 (5,000–10,000 IU daily) to the patients diets but cautioned that controlled trials were needed to evaluate this therapeutic strategy. Their hypothesis was given credence by observations made by Saied et al. who suggested that the wide traditional use of medicinal plants as hot or cold beverages, and as flavours added to foods played a role in mitigating COVID-19 symptoms in Egyptian patients.

COVID-19 has been associated with cold-dampness, often described as a feeling of heaviness, puffiness of the skin, phlegm discharge and water retention in the abdomen, etc. Cold-dampness is an etiological factor in Chinese medicine (TCM). Zheng et al. reviewed a series of TCMs—Huoxiang Zhengqi Dropping Pills, Lianhua Qingwen Granules Hanshiyi Formula, and Tongzhi Granule that were integral to the treatment of COVID-19 in Wuhan City; Despite their clinical efficacy and therapeutic pharmacology speculation, the authors suggested large-scale randomized clinical trials, cell and animal experiments were needed to further verify the theory of the Cold-dampness Plague in COVID-19. In a related study, Wegbom et al. conducted a population-based survey in Nigeria to estimate the knowledge level, causes, prevalence, and determinants of self-medication practices for the prevention and/or treatment of COVID-19. The authors observed that among the 461 respondents, 41% reported using drugs such as vitamin C and multivitamin (51.8%), and antimalarials (24.9%) for self-medication of COVID-19. Among these self-medicated patients, 79.5% and 77.3% reportedly self-medicated due to fear of stigmatization or discrimination and fear of being quarantine, respectively. Based on their findings, the authors proposed a number of interventions including media and community-based educational campaigns to raise awareness about the dangers of self-medication for COVID-19.

### Genomic surveillance and evolution of SARS-CoV-2

The COVID-19 pandemic has spurred the development of innovative technologies and ways that pathogen genomic surveillance can be used to monitor outbreaks and track infected individuals and their close contacts. Briefly, the majority of SARS-CoV-2 genomic surveillance studies that were published in this Research Topic utilized whole genome sequences and phylogenetic approaches to monitor the transmission dynamics and evolution of the virus in different geographical regions across multiple timepoints.

Henriques-Santos et al. used SNP assays to genotype SARS-CoV-2 infections in samples from industry workers in Rio de Janeiro, Brazil. The authors demonstrated that the Zeta variant (variant of interest or VOI) had been in circulation since October 2020 and reached 87% prevalence in February 2021 followed by a decrease due to emergence of the Gama variant, a variant of concern or VOC. Fan et al. analysed 1,051 near-complete and 1,559 spike sequences belonging to the B.1.617 lineage (GISAID database). They identified 22 positive selection sites in the genome, mostly distributed across the spike glycoprotein.

Several reports indicate that SARS-CoV-2 has evolved multiple times and independently in different geographical regions. Laiton-Donato et al. were the first to report 21 distinctive mutations, including L249S and E484K in the spike gene of Colombian isolates while Narayanan et al. reported mutations including D614G in S gene, P4715L in ORF1ab, S194L, R203K and G204R in N gene, in isolates from Oklahoma, United States. Yi et al. analyzed publicly available genomes in GISAID and PANGO and suggested that the B.1.1.7, B.1.351, and P.1 lineages were more transmissible than variants harbouring key mutations in the spike glycoprotein—N501Y, E484K, and K417N/T. The latter findings were corroborated by Akkiz who reported similar mutations in global isolates. Based on his findings, the author suggested that some SARS-CoV-2 vaccines may be less efficacious against the B.1.351 lineages (Akkiz). This vaccine inefficacy assertion was further supported with homology modelling data generated by Zhang et al. who conducted a comprehensive analysis of >300 million peptides derived from 13,432 SARS-CoV-2 strains that harboured 4,420 amino acid mutations. In their analysis, protein docking simulations indicated that mutations in spike glycoprotein may enhance some SARS-CoV-2 variants to invade host cells and escape antibody-mediated B-cell immunity.

Source-tracking SARS-CoV-2 infections using both genomic and epidemiological data has been key in contact-tracing efforts and in the identification of local outbreaks. Zhu et al. estimated that the time to the most recent common ancestor (tMRCA) of SARS-CoV-2 circulating in Southeast Asia was 28 November 2019 (95% confidence interval (CI) 7 September 2019 to 4 January 2020). This tMRCA was associated with an evolutionary rate of 1.446 × 10^−3^ (95% CI: 1.292 × 10^−3^ to 1.613 × 10^−3^) substitutions per site per year. The authors suggested that Singapore and Thailand were the two most probable root positions, i.e., tMRCA.

Among European variants, phylogenetic analysis of Romanian genomes (*n* = 112) with sequences originating from Europe, United Kingdom, Africa, Asia, South and North America (*n* = 876) indicated multiple introduction events for SARS-CoV-2 in Suceava, Romania. These introductions were traced to Italy, Spain, United Kingdom and Russia although some sequences were also related to those from the Czech Republic, Belgium, and France (Lobiuc et al.). Early diffusion of SARS-CoV-2 infection in the inner areas of the Italian Sardinia island was associated with imported cases (Piras et al.). Zrelovs et al. presented an investigative high-throughput framework utilised by the Latvian Biomedical Research and Study Center for tracking outbreaks. The authors appraised the strategy of linking epidemiological data with genetic makeup of priority isolates being sequenced to aid epidemiological investigations.

### Sero-surveillance and immunity

Anti-SARS-CoV-2 antibodies reportedly can last over 12 months following an infection. Xiao et al. followed 56 COVID-19 survivors to assess how long antibodies acquired to SARS-CoV-2 persisted. The authors showed that immunoglobulin G (IgG) titres decreased significantly in the first 6 months and remained stable in the following 6 months (Xiao et al.). Tomas-Grau et al. investigated anti-RBD (receptor binding domain) antibodies among individuals in the province of Tucumán, Argentina. They showed that individuals from a high altitude village displayed elevated and longer lasting antibody titres compared to those from a lower altitude city. Acquisition of IgG antibodies following infection was also confirmed among residents in Lisbon, Portugal. However, in the latter cohort, the authors could not detect anti-spike IgA antibodies (Gonçalves et al.). Tang et al. showed, in a systematic review, that re-detectable SARS-CoV-2 in patients who had recovered from COVID-19 was plausibly due to reactivation, reinfection, viral shedding, or testing errors. Their data further suggested that acquisition of immunity following previous exposure may not prevent reinfection.

### Host-genetics and COVID-19

Host genetic traits including red blood cell (RBC) phenotypes—ABO were associated with COVID-19 clinical forms. Allelic variants within the ABO blood group were associated with protection against critical COVID-19 symptoms. In a cross-sectional study involving 646 SARS-CoV-2-positive participants from multiple hospitals and population-based (quarantine camps) recruitment sites in the United Arab Emirates, Jelinek et al. showed that patients with blood group O were less likely to develop symptoms of critical COVID-19 (odds ratio: 0.51 (95% CI: 0.33, 0.79); *p*-value = 0.003). The protective effect in this blood group was associated with eight alleles in the 3′untranslated region (UTR) of chromosome 9: rs199969472, rs34266669, rs76700116, rs7849280, rs34039247, rs10901251, rs9411475, and rs13291798. In a retrospective familial cluster study using data from the Centre for Disease Control (CDC), Liu et al. compared the clinical features and computer tomography (CT) changes between familial clusters (i.e., ≥2 COVID-19 patients in a family, *N* = 108) and non-familial patients (*N* = 70) with COVID-19 pneumonia. The authors observed that patients in the familial cluster had a worse clinical course and outcome (prolonged virus shedding period, longer hospital stay and slower resolution of lung abnormalities) than those in the non-familial cluster. These poor outcomes were associated with lower lymphocyte counts in the familial cluster group, further underscoring the role of host factors in COVID-19 severity.

### SARS-CoV-2 infection models

A number of animal models including hamster, mouse, ferret, mink, tree shrew and nonhuman primate models have been reported as infection models for studying the virology of SARS-CoV-2 (Shou et al.). Varotto-Boccazzi et al. demonstrated the utility of *Leishmania tarentolae* as an easy-to-handle tool to produce antigens for viral diagnosis. The authors engineered *L. tarentolae* to express the SARS-CoV-2 RBD, which were purified and used to detect SARS-CoV-2 infection in human sera. The sensitivity and reproducibility of the *L. tarentolae* assay was comparable to that of a reference antigen produced in human cells.

### Modelling the COVID-19 epidemic

Modelling of COVID-19 data from different epidemiological settings and clinical cases has provided key statistical estimations of risk of infection, which has informed public health control efforts. Sun et al. established a mathematical model using epidemiological data and global sensitivity analysis method to identify age and clinical parameters—body temperature, levels of troponin and blood glucose as associates of mortality in COVID-19 patients with hypotension and hypoxemia. The authors asserted the utility of their model to effectively describe and predict the evolution of the epidemic in the United States. Tan et al. applied a Mendelian randomization (MR) design to show that hospitalized COVID-19 cases had an increased risk of ischemic stroke. Other models including Kaplan-Meier type survival curves for COVID-19 were proposed by Calabuig et al. for a health data-based decision-making tool to provide useful information about the dynamics of the disease in different countries.

### Software programs for tracking cases

Accurate count of daily COVID-19 cases has been vital to public health control interventions, particularly, in identifying transmission hotspots and in allocating resources for control interventions. Salehi et al. developed a synergetic R-Shiny portal, with ac R-Shiny portal, with an interactive web interface. The authors demonstrated the utility of this application for modelling and real-time tracking of confirmed cases, recoveries and deaths due to COVID-19, at the national and continental levels.

### Control and prevention

The epidemiological risk of SARS-CoV-2 infection and of COVID-19 disease has been reported within and across different countries, each with different control strategies. In the Hubei Province of China, Ren et al. conducted a retrospective cross-sectional study between 8 and 20 February 2020, involving 289 COVID-19 patients from three regions; They showed that there were regional differences in the burden of COVID-19 in the three regions—Urban (Wuhan Union West Hospital), Suburban areas of Wuhan (Hannan Hospital) and Enshi city. Hu et al. found that in China, during 2021, public health measures such as wearing face mask, physical distancing and isolation during the COVID-19 pandemic significantly (*p*-value < 0.05) reduced the spread of other respiratory infectious diseases such as measles, tuberculosis, pertussis, scarlet fever, influenza and mumps. Li et al. used an age-structured model to show that COVID-19 incidence and deaths, respectively, could have been significantly reduced in New York state (United States) If all infected people were diagnosed and hospitalized in time.

## Conclusion

We believe that the findings presented in this editorial and in the articles published in the Research Topic has contributed to our understanding of the origin of the virus, its evolution and transmission dynamics among different populations in different geographical regions. Additionally, we hope that the data presented in the articles published in this Research Topic will have wide applicability across other infectious disease areas—surveillance, diagnosis, treatment and public health control interventions of the COVID-19 pandemic.
